# Achalasia—An Autoimmune Inflammatory Disease: A Cross-Sectional Study

**DOI:** 10.1155/2015/729217

**Published:** 2015-05-20

**Authors:** J. Furuzawa-Carballeda, D. Aguilar-León, A. Gamboa-Domínguez, M. A. Valdovinos, C. Nuñez-Álvarez, L. A. Martín-del-Campo, A. B. Enríquez, E. Coss-Adame, A. E. Svarch, A. Flores-Nájera, A. Villa-Baños, J. C. Ceballos, G. Torres-Villalobos

**Affiliations:** ^1^Department of Immunology and Rheumatology, National Institute of Medical Sciences and Nutrition, Vasco de Quiroga No. 15, Colonia Sección XVI, 14000 Mexico City, DF, Mexico; ^2^Department of Pathology, National Institute of Medical Sciences and Nutrition, Vasco de Quiroga No. 15, Colonia Sección XVI, 14000 Mexico City, DF, Mexico; ^3^Department of Gastroenterology, National Institute of Medical Sciences and Nutrition, Vasco de Quiroga No. 15, Colonia Sección XVI, 14000 Mexico City, DF, Mexico; ^4^Department of Experimental Surgery, National Institute of Medical Sciences and Nutrition, Vasco de Quiroga No. 15, Colonia Sección XVI, 14000 Mexico City, DF, Mexico

## Abstract

Idiopathic achalasia is a disease of unknown etiology. The loss of myenteric plexus associated with inflammatory infiltrates and autoantibodies support the hypothesis of an autoimmune mechanism. Thirty-two patients diagnosed by high-resolution manometry with achalasia were included. Twenty-six specimens from lower esophageal sphincter muscle were compared with 5 esophagectomy biopsies (control). Immunohistochemical (biopsies) and flow cytometry (peripheral blood) analyses were performed. Circulating anti-myenteric autoantibodies were evaluated by indirect immunofluorescence. Herpes simplex virus-1 (HSV-1) infection was determined by* in situ *hybridization, RT-PCR, and immunohistochemistry. Histopathological analysis showed capillaritis (51%), plexitis (23%), nerve hypertrophy (16%), venulitis (7%), and fibrosis (3%). Achalasia tissue exhibited an increase in the expression of proteins involved in extracellular matrix turnover, apoptosis, proinflammatory and profibrogenic cytokines, and Tregs and Bregs* versus *controls (*P* < 0.001). Circulating Th22/Th17/Th2/Th1 percentage showed a significant increase* versus *healthy donors (*P* < 0.01). Type III achalasia patients exhibited the highest inflammatory response* versus *types I and II. Prevalence of both anti-myenteric antibodies and HSV-1 infection in achalasia patients was 100%* versus *0% in controls. Our results suggest that achalasia is a disease with an important local and systemic inflammatory autoimmune component, associated with the presence of specific anti-myenteric autoantibodies, as well as HSV-1 infection.

## 1. Introduction

Idiopathic achalasia is one of the most common causes of esophageal motor disorders. Manometrically, it is characterized by aperistalsis, failure of lower esophageal sphincter (LES) relaxation in response to swallows, and increased LES resting tone. Clinical manifestations include dysphagia to solids and liquids but the patients may also have regurgitation of esophageal contents, chest pain, cough, aspiration, weight loss, and heartburn [1, 2]. Achalasia affects both sexes and all age groups. The prevalence of the disease is eight cases per million population with an estimate incidence of approximately 0.3–1.63 per 100,000 habitants per year [3, 4]. Diagnosis is established by high-resolution manometry (HRM) as the gold standard while the esophagram with barium swallow and endoscopy is useful to rule out other pathologies. Idiopathic achalasia has been classified in three types based on the HRM: achalasia with minimal esophageal pressurization (type I), achalasia with esophageal compression (type II), and achalasia with spasm (type III) [5].

Even though the disease was first described more than 300 years ago, etiology and physiopathology are still unknown. However, many contemporary hypotheses suggest a multifactorial etiopathogenic mechanism [6, 7]. Thus, pathophysiologically, idiopathic achalasia is caused by loss of inhibitory ganglion in the myenteric plexus of the esophagus [7–11]. In order to explain this, several studies have attempted to explore initiating agents that may cause the disease, such as latent or active viral infection. Proposal virus candidates have included herpes simplex virus (HSV) (neurotropic virus with a predilection for squamous epithelium), bornavirus, varicella zoster (VZ), measles, and human papilloma virus (HPV) [12, 13]. Nonetheless, not all the patients with viral infections develop the disease. Numerous potential genetic targets exist in the immune system, which may create susceptibility. Some studies have been shown genetic correlations in achalasia, for example, ALADIN gen associated with Allgrove syndrome, receptor of vasoactive intestinal polypeptide (VIPR1) gene polymorphism, interleukin- (IL-) 23 receptor polymorphism, protein tyrosine phosphatase nonreceptor 22 gene polymorphism, and human leukocyte antigen- (HLA-) DQ*β*1 (*HLA*-*DQB*1^*∗*^ *05:03*  and *HLA*-*DQB*1^*∗*^ *06:01*), HLA-DQ*α*1 (*HLA*-*DQA*1^*∗*^ *01:03)*, and HLA-DQ*β*1 (*HLA*-*DQB*1^*∗*^ *03:01 *and *HLA*-*DQB*1^*∗*^ *03:04)* [8]. Consequently, chronic viral infection could trigger aberrant immune response under appropriate genetic and environmental background allowing the loss of esophageal neurons. Moreover, detailed examination of Auerbach's plexus has shown infiltration of CD3^+^/CD45RO^+^ T cells, predominantly CD8^+^ T cytotoxic lymphocytes expressing activation markers [7, 10, 11, 14]. Notwithstanding, little is known about whether these subpopulations of cells are truly autoimmune, as in the case of pathogenic Th22 and Th17 effector cell subsets, and what happens with cellular mechanisms which are participating in an attempt to regulate the inflammation, such as regulatory CD4^+^ T cells and IL-10-producing B cells [15, 16]. Finally, in accordance with the hypotheses, evidence autoantibodies against myenteric neurons have been shown in serum samples from patients with achalasia, especially in those with HLA  DQA1^*∗*^0103 and DQB1^*∗*^0603 alleles [17]. However, no specific antigenicity of myenteric neuron autoantibodies has been identified [2, 18].

Based on these data and hypotheses, the aim of the study was to analyze in esophageal muscle tissue and peripheral blood the localized and systemic inflammation patterns in idiopathic achalasia patients. Furthermore, we evaluate the existence of circulating anti-myenteric autoantibodies and their specificities in sera from patients and assess the presence of herpes simplex virus type 1 (HSV-1) in esophageal muscle.

## 2. Materials and Methods

### 2.1. Study Design

This was an exploratory, observational, and cross-sectional study designed to identify by the first time the presence of CD4^+^ T-cell subpopulations related to autoimmune diseases, autoantibodies and their specificity and proteins involved in extracellular matrix turnover, apoptosis, proinflammatory and profibrogenic cytokines, and regulatory T and B cells (Tregs and Bregs) in lower esophageal sphincter biopsies from achalasia patients. Thirty-two patients with idiopathic achalasia without Chagas disease, HIV, or HCV infections were included between January 2013 and July 2014. The medical records of each patient were reviewed. Peripheral blood was obtained from 20 age-matched healthy donors (HD) who volunteered at the blood bank. HDs were also interviewed in order to discard any disease including known autoimmune disease, use of immunosuppressors or prednisone, and concurrent infections. Five biopsies of esophagectomy from patients with squamous cell carcinoma from upper third of esophagus were included as control tissue samples (see Supplementary Figure 1 in Supplementary Material available online at http://dx.doi.org/10.1155/2015/729217).

### 2.2. High Resolution Esophageal Manometry Protocol

A high-resolution esophageal manometry was performed in every patient at baseline and before being referred to surgery. A solid-state HRM probe with 36 circumferential sensors was used (Given Imaging, Yokneam, Israel). Having the patient in a sitting position and at 45 degrees, stationary HMR was performed. After a 12 hr fasting period, the probe was inserted transnasally until passing the esophagogastric junction, assessed visually in the computer screen. Ten water swallows of 5 mL, separated by 30 seconds were provided.

Analyses were performed using Manoview 2.0 (Given Imaging) and patients were classified according to latest Chicago classification [19] into three groups: (1) type I achalasia (without pressurization within the esophageal body), (2) type II (with pan-pressurization), and (3) type III (with spasm). Classification was performed by two gastroenterologists (MAV, E-CA) experts in high-resolution esophageal manometry. Surgeon was blinded to the high-resolution manometry analysis [19].

### 2.3. Erythrocyte Sedimentation Rate

Erythrocyte sedimentation rate (ESR) was determined by Westergren method.

### 2.4. Immunohistochemistry

Once the myotomy was done, a 2 cm long and 2 mm width tissue was obtained. Tissue was immediately formalin-fixed and paraffin embedded. Four *μ*m thick tissue sections were deparaffinized and rehydrated. Tissues were incubated for 18 h at 4°C with goat polyclonal anti-human MMP-9, TIMP-1, TGF-*β*1, IL-22, IL-17A, FAS, or mouse monoclonal IgG_1_ HSV-1 antibody (Santa Cruz Biotechnology, Santa Cruz, CA, USA) at 10 *μ*g/mL. Binding was detected by incubating sections with biotinylated donkey anti-goat IgG antibody (ABC Staining System; Santa Cruz Biotechnology). Slides were incubated with horseradish peroxidase- (HRP-) streptavidin and 3,3′-diaminobenzidine (DAB) (Sigma-Aldrich) and counterstained with hematoxylin. Negative control staining was performed with normal human serum diluted 1 : 100, instead of primary antibody. The reactive blank was incubated with phosphate buffer saline-egg albumin (Sigma-Aldrich) instead of the primary antibody. Both controls excluded nonspecific staining or endogenous enzymatic activities. At least two different sections and two fields (×320) were examined for each biopsy. Immunostained cells were assessed by estimating the number of positively staining cells in two fields (×320) and were reported as the percentage of immunoreactive cells. Results are expressed as the mean ± standard error of the mean (SEM) of cells quantified by the program Image Pro Plus version 5.1.1 [15].

### 2.5. Double-Staining Procedure

To determine the subpopulation of IL-13^+^/IL-4^+^-, CD4^+^/IFN-*γ* ^+^-expressing T cells, CD25^+^/Foxp3^+^ regulatory T cells, and CD20^+^/IL-10^+^-producing B-cell subpopulations a simultaneous detection was performed [EnVision G|2 Doublestain System (Dako, Glostrup, Denmark)]. The procedure is a sequential double staining where the first antigen was visualized using HRP/3′3′-diaminobenzidine (DAB) and the second antigen was visualized using alkaline phosphatase (AP)/permanent red. Briefly, incubation of samples with 200 *μ*L of dual endogenous enzyme block was performed. This procedure inhibited endogenous AP, peroxidase and pseudoperoxidase activity present in tissues. After blocking, tissues were incubated with 200 *μ*L of normal serum as negative control or second primary rabbit polyclonal anti-CD20, anti-CD25 IgG antibody, or mouse monoclonal anti-CD4 or anti-IL-4 IgG_1_ antibody (Santa Cruz Biotechnology) at 10 *μ*g/mL. Then 200 *μ*L of rabbit/mouse LINK was added for 10 min. Tissues were incubated with 200 *μ*L of polymer/AP reagent. Reaction was visualized by incubation with 200 *μ*L permanent red chromogen. Tissues were counterstained with hematoxylin and mounted in aqueous mounting medium. At least two different sections and two fields (×320) were examined for each biopsy. Double positive IL-4^+^/IL-13^+^-, CD4^+^/IFN-*γ* ^+^-expressing T cells, CD25^+^/Foxp3^+^ regulatory T cells, and CD20^+^/IL-10^+^-producing B cells were assessed by estimating positively staining cells and results were reported as the percentage of immunoreactive cells. Results are expressed as the mean ± SEM of cells quantified by the program ImagePro Plus version 5.1.1 [16].

### 2.6. Flow Cytometry

A sample of venous blood (10 mL) was obtained from each subject. Peripheral blood mononuclear cells (PBMCs) were isolated by gradient centrifugation on Lymphoprep (Axis-Shield PoC AS, Oslo, Norway).

For Th22 (CD3^+^/CD4^+^/CD161^−^/IL-22^+^), Th17 (CD3^+^/CD4^+^/CD161^+^/IL-17A^+^), Th2 (CD3^+^/CD4^+^/CD14^−^/IL-4^+^), Th1 (CD3^+^/CD4^+^/CD14^−^/IFN-*γ* ^+^), and regulatory T cells (CD3^+^/CD4^+^/CD25^+^/Foxp3^+^), PBMCs were labelled with 5 *μ*L of anti-human CD3-FITC-, anti-CD4-PeCy5-, and anti-CD161-APC-conjugated monoclonal antibodies (BD Biosciences, San Jose, CA); anti-CD14-FITC-, anti-CD4-PeCy5-, anti-CD3-APC- or CD3-FITC-, anti-CD4-PeCy5-, and anti-CD25-APC-conjugated monoclonal antibodies (BD Biosciences, San Jose, CA). Intracellular staining was performed with 5 *μ*L of an anti-human IL-22-PE-, IL-17A-PE-, IL-4-PE-, IFN-*γ*-PE-, or anti-human Foxp3-PE-labelled monoclonal antibodies (BD Biosciences). Finally, T subsets were analyzed by flow cytometry with an Accuri C6 (BD Biosciences). A total of 500,000–1,000,000 events were recorded for each sample and analyzed with the FlowJo X software (Tree Star, Inc.). An electronic gate was made for CD3^+^/CD4^+^/CD161^−^, CD3^+^/CD4^+^/CD161^+^, CD3^+^/CD14^−^/CD4^+^ cells, or CD3^+^/CD4^+^/CD25^hi^. Results are expressed as the relative percentage of IL-22-, IL-17A-, IL-4-, IFN-*γ*-, or Foxp3-expressing cells in each gate.

As isotype control, IgG_1_-FITC/IgG_1_-PE/CD45-PeCy5 mouse IgG_1_,*κ* (BD Tritest, BD Biosciences) was used to set the threshold and gates in the cytometer. We ran an unstained (autofluorescence control) and permeabilized PBMCs sample. Autofluorescence control was compared to single stained cell positive controls to confirm that the stained cells were on scale for each parameter. Besides, BD Calibrite 3 beads were used to adjust instrument settings, set fluorescence compensation, and check instrument sensitivity (BD CaliBRITE, BD Biosciences). Fluorescence minus one (FMO) controls were stained in parallel using the panel of antibodies with sequential omission of one antibody [20].

### 2.7. Circulating Neurologic Autoantibodies

Serum antibodies were evaluated with a commercially available kit Neurology Mosaic 1 (Euroimmun, Lübeck, Germany) by standard indirect immunofluorescence screening assay using as antigenic substrate frozen monkey nerves, cerebellum, and intestinal tissue, following manufacturer's instructions. Sera from patients and controls diluted at 1/10 were incubated on intestinal, cerebellum, and peripheral nerves of monkey tissue sections. A positive anti-Hu serum was used as control. Fluorescein-labelled anti-human immunoglobulin G (IgG) conjugate was used as secondary antibody. Slides were examined on a fluorescence photomicroscope (×200 and ×400). Anti-Hu-positive serum labelled neuron nuclei on both cerebellum and myenteric plexus sections. For circulating neurologic antibodies positivity staining of both nucleus and cytoplasm of myenteric plexus neurons was considered [21].

### 2.8. Immunoblot Analysis

To further analyze target antigens of circulating anti-myenteric autoantibodies, sera were tested with the neuronal antigens profile plus RST (Euroimmun). This test is a membrane strip with a combination of neuronal antigens (amphiphysin, CV2, and PNMA2 (Ma-2/Ta)), onconeural antigens (Ri, Yo, and Hu), recoverin, SOX-1, and titin separately. After blot strip blocking, sera were incubated at 1/100 for 1 hour at room temperature. To detect the bound antibodies, a second incubation was carried out using alkaline phosphatase-labelled antihuman IgG. For the interpretation a EUROLine Scan software (Euroimmun) was used [21].

### 2.9. *In Situ* Hybridization for HSV-1

Paraffin-embedded specimens of lower esophageal sphincter muscle from achalasia patients and controls were deparaffinized for 18 h at 60°C and sequentially immersed in xylene (30 min at 37°C), absolute ethanol, 75% ethanol, 50% ethanol, 25% ethanol, and water. Cells were permeabilized and incubated with 1 mg/L proteinase K (Promega, Madison, WI, USA) for 30 min at 37°C. Codenaturation and hybridization were done by separation of double-strand DNA and binding of single-stranded probe. HSV-1 FITC-conjugated PNA probe mixture (Dako, Denmark) was applied to the specimen on each slide and they were sealed and incubated for 3 min at 73°C, then for 4 hrs at 37°C and finally for 2 hrs at 55°C. Probe detection was done by the addition of an anti-FITC/AP antibody and alkaline phosphatase. Positive cells were considered if the purple reaction product was seen. Tissues were counterstained with nuclear fast red. The negative controls included 5 biopsies of esophagectomy from patients with squamous cell carcinoma and were identified as noninfected patients (negative for HSV-1 DNA).

### 2.10. RT-PCR for HSV

Detection of HSV-1 DNA was performed by reverse transcriptase and PCR in specimens from lower esophageal sphincter muscle from achalasia patients and controls. Four *μ*m thick sections of available formalin-fixed paraffin embedded tissue were deparaffinized and rehydrated. Cells were permeabilized and incubated with 1 mg/L proteinase K (Promega, Madison, WI, USA) for 30 min at 37°C. Tissue was treated with DNase I (Invitrogen, Alameda, CA, USA) and slides were sealed with the assembly tool and placed in a thermocycler (Perkin Elmer, Cambridge, UK). RT was done by incubation of the sections with 50 ng random primers, 1 *μ*L 10 mM dNTPs, water 15 *μ*L, 4 *μ*L 5x first-strand buffer, 2 *μ*L 0.1 M DTT, 1 *μ*L RNase OUT, and 200 U of M-MLV RT (Invitrogen, Alameda, CA, USA) in a thermocycler (Perkin Elmer) at 37°C for 50 min. PCR reaction was performed with 1x reaction buffer (Invitrogen, Alameda, CA, USA), 1.5 U Taq polymerase, 2 mmol/L MgCl_2_, 40 mmol/L dNTP, 0·2 mmol/L dUTP-digoxigenin (Boehringer Mannheim, Lewes, UK), and 60 pg each of HSV-1 primers (5′ CATCACCGACCCGGAGAGGGAC3′ (sense) and 5′GGGCCAGGCGCTTGTTGGTGTA3′ (antisense)). The slides were sealed with the assembly tool (Perkin Elmer) and placed in a Touchdown Thermocycler (Perkin Elmer, Cambridge, UK). cDNA was amplified as follows: denaturation at 95°C for 1 min, annealing at 70°C for 1 min, and extension at 72°C for 1 min, for 35 sequential cycles. PCR products were detected with alkaline phosphatase-conjugated sheep antibodies against digoxigenin (Roche Mannheim Germany) diluted 1/500 and the chromogen was 5-bromo-4-chloro-3-3 indolyl phosphate toluidine salt and tetrazolium nitroblue (Boehringer Manheim) diluted 1/50. Sections were counterstained with nuclear fast red or malachite green to avoid any interference with the blue signal generated by HSV DNA. To avoid bias, one section from one block from each individual was used in masked assays and it was scored positive if the blue reaction product was seen. One section from a patient with infection HSV-1 was used as positive control. The negative controls included 5 biopsies of esophagectomy from patients with squamous cell carcinoma and were identified as noninfected patients (negative for HSV-1 DNA).

### 2.11. Statistical Analysis

Descriptive statistics were performed and categorical variables were compared using *X* ^2^ test or Fisher's exact test; for analysis of continuous variables Student *t*-test was employed. One-way analysis of variance on ranks by Holm-Sidak method was performed for both pairwise comparisons and comparison versus a control group. Statistical analysis was done using the SigmaStat11.2 program (Aspire Software International, Leesburg, VA, USA). Data were expressed as the median, range, and mean ± standard deviation (SD)/standard error of the mean (SEM). The *P* values smaller than or equal to 0.05 were considered as significant. This study conforms to STROBE statement along with references to STROBE and the broader EQUATOR guidelines [22].

### 2.12. Study Approval

This work was performed according to the principles expressed in the Declaration of Helsinki. The study was approved by the ethical committee from our institution and a written informed consent was obtained from all subjects.

## 3. Results

### 3.1. Demographic Data

The study included 32 patients with idiopathic achalasia. Twenty-five percent of patients were type I, 59% type II, and 16% type III achalasia. The mean age of the patients was similar among the 3 types. Although the female to male ratio was similar in type I (4/4), there was a difference in sex ratio in types II (12/7) and III (4/1). Two patients from type II achalasia group also had Sjögren's Syndrome and Guillain-Barre Syndrome, respectively. Two patients from type III achalasia group also had Sjögren's Syndrome and ankylosing spondylitis. Demographic and clinical variables and laboratory data from the healthy donors (PBMCs), tissue control group (esophageal biopsies), and the achalasia patients are described in Table 1.

### 3.2. Histological Findings

**Figure 1:**
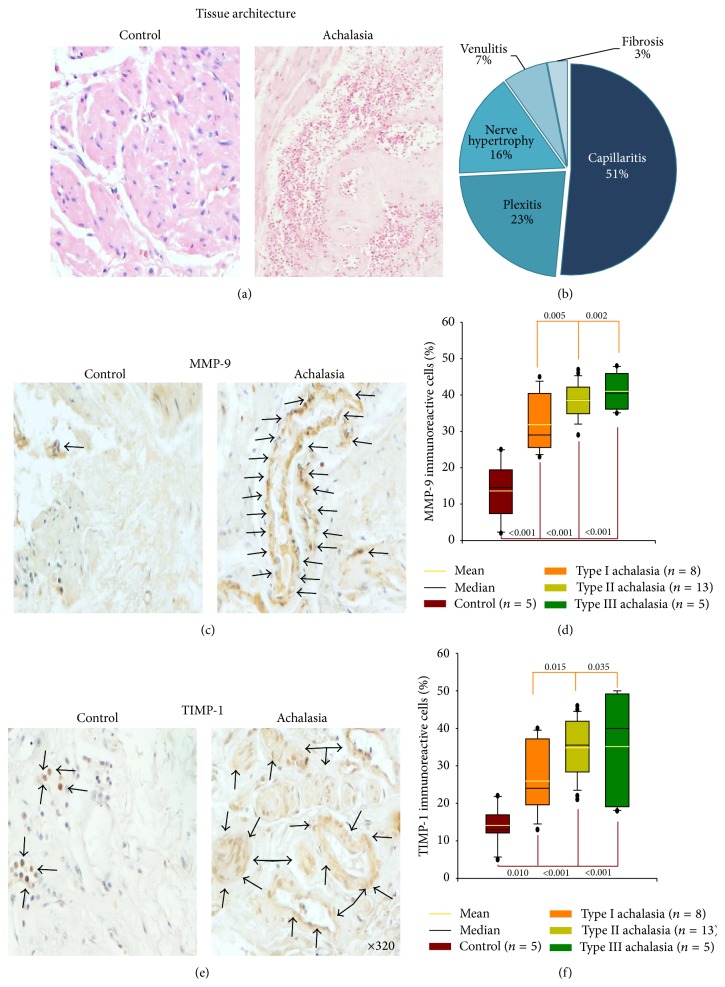
Proteins involved in extracellular matrix turnover in achalasia. (a) Representative photomicrograph from a control and an idiopathic achalasia tissue in which an abundant inflammatory infiltrate is observed. Original magnification was ×200. (b) Histological findings in biopsies from achalasia patients. (c) Immunoreactive cells. Original magnification was ×320. (d) Results are expressed as mean (yellow line), median (black line), and 5th/95th percentiles of MMP-9^+^ cells. (e) Immunohistochemistry for TIMP-1 in control and achalasia sections. Arrows depict immunoreactive cells. Original magnification was ×320. (f) Results are expressed as mean (black line), median (yellow line), and 5th/95th percentiles of TIMP-1^+^ cells.

Esophageal muscle biopsies from primary achalasia patients had abundant perineural inflammatory infiltrates, as well as an increase of endoneurial connective tissue and myopathic changes. Histopathological analysis showed capillaritis (51%), plexitis (23%), venulitis (7%), fibrosis (3%), and nerve hypertrophy (16%) (Figures 1(a) and 1(b)).

### 3.3. Proteins Involved in Extracellular Matrix Turnover

MMP-9 also known as 92 kDa gelatinase is involved in extracellular matrix degradation in physiological and pathological processes. The myenteric plexus of esophageal tissue from achalasia patients showed a statistically significant increase in MMP-9 percentage versus tissue control group. Nonetheless, the number of MMP-9^+^ cells in types II and III achalasia was higher when compared to type I achalasia (Table 2; Figures 1(c) and 1(d)).

TIMP-1 inhibits not only the proteolytic activity but also apoptosis and induces cell proliferation. TIMP-1^+^ cell percentage was higher in achalasia patients compared with control group. Moreover, number of TIMP-1-producing cells in types II and III achalasia was higher when compared to type I achalasia (Table 2; Figures 1(e) and 1(f)).

### 3.4. Expression of Pro-Inflammatory/Anti-Fibrogenic Cytokines

**Figure 2:**
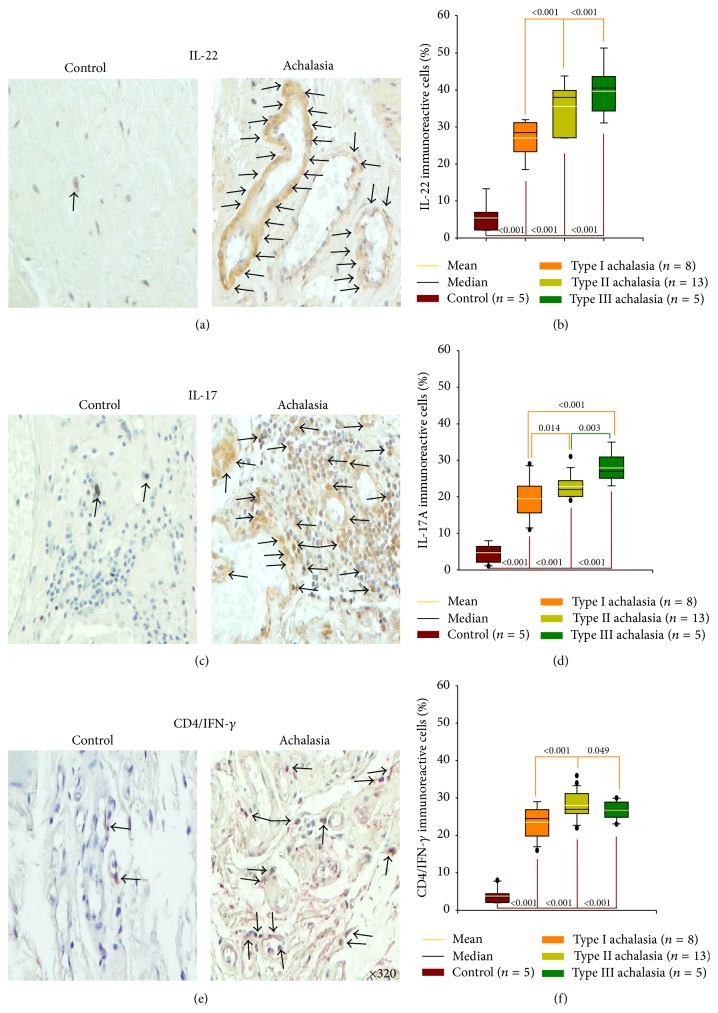
Proinflammatory/antifibrogenic cytokine expression in achalasia. Immunohistochemistry for (a) IL-22, (c) IL-17, and (e) CD4/IFN-*γ* T cells in control and achalasia sections. Arrows depict immunoreactive cells. Original magnification was ×320. Results are expressed as mean (yellow line), median (black line), and 5th/95th percentiles of (b) IL-22^+^, (d) IL-17^+^, and (f) CD4/IFN-*γ* ^+^ T cells.

IL-22 belongs to the IL-10 superfamily. It initiates innate immune response against pathogens in gut epithelial and respiratory cells and regulates antibody production. IL-22^+^ cell percentage was conspicuously increased in myenteric plexus of esophageal tissue samples when compared with control group. However, IL-22^+^ cell number was increased in type II and type III achalasia versus type I achalasia (Table 2; Figures 2(a) and 2(b)).

IL-17 is defined as key mediator of inflammatory autoimmune diseases. It promotes recruitment of neutrophils, activation of innate immune cells, enhances B-cell functions, and induces proinflammatory cytokines (TNF-*α*, IL-1*β*). IL-17A^+^ cell frequency was higher in myenteric plexus of esophageal achalasia tissue patients versus control tissue. Moreover, a higher immunoreactive cell number was found in types II and III achalasia when compared with type I achalasia (Table 2; Figures 2(c) and 2(d)).

Achalasia patients had significantly higher percentage of IFN-*γ*/CD4 T cells versus control group. Nonetheless, levels of IFN-*γ*/CD4 T cells were higher in types II and III achalasia compared with type I achalasia (Table 2; Figures 2(e) and 2(f)).

### 3.5. Expression of Anti-Inflammatory/Profibrogenic Cytokines

**Figure 3:**
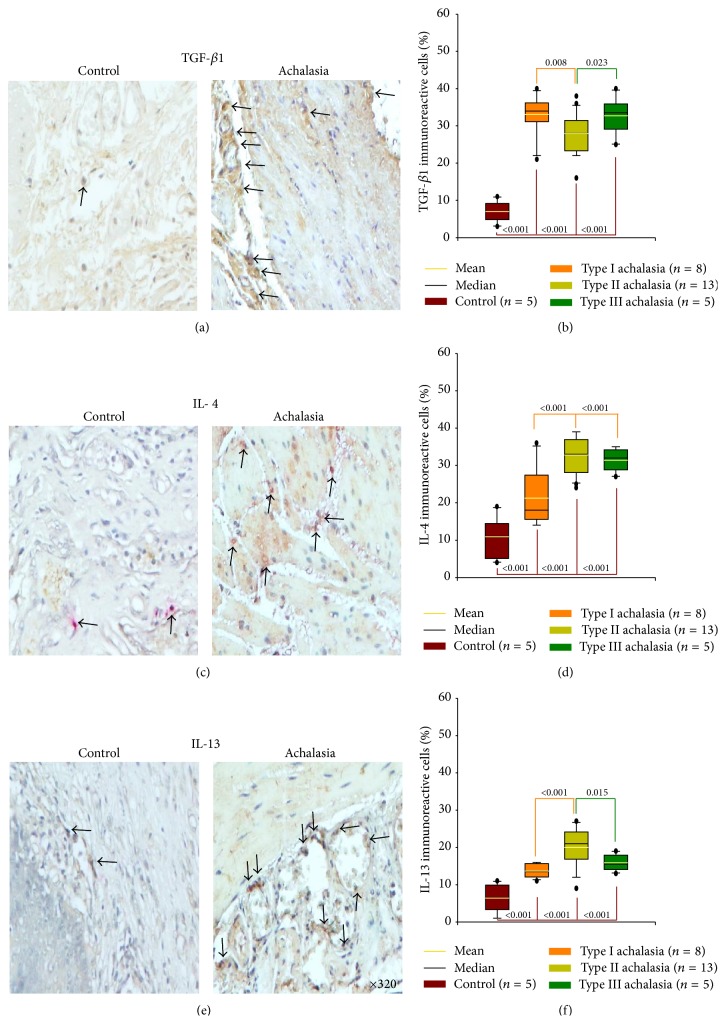
Anti-inflammatory/profibrogenic cytokine expression in achalasia. Immunohistochemistry for (a) TGF-*β*1, (c) IL-4, and (e) IL-13 cells in control and achalasia sections. Arrows depict immunoreactive cells. Original magnification was ×320. Results are expressed as mean (yellow line), median (black line), and 5th/95th percentiles of (b) TGF-*β*1^+^, (d) IL-4^+^, and (f) IL-13^+^ cells.

TGF-*β*1 accomplishes many cellular functions including control of cellular growth, proliferation, differentiation, negative regulation of inflammation, collagen synthesis, and apoptosis. Thus, myenteric plexus of esophageal biopsies from idiopathic achalasia patients showed a significant increase on TGF-*β*1^+^ cell expression compared with control tissue. It is important to highlight that types I and III achalasia patients had higher percentage of immunoreactive cells versus type II (Table 2; Figures 3(a) and 3(b)).

IL-4 is an anti-inflammatory cytokine that inhibits the synthesis of IL-1*β*, TNF-*α*, IL-6, IL-17A, and so forth. It regulates B-cell proliferation and differentiation, and it is also a potent inhibitor of apoptosis. IL-4 is synthesized primarily by Th2 cells. Achalasia patients had significantly higher IL-4^+^ cell percentage when compared with control group. However, IL-4^+^ cell number was higher in type II and type III achalasia versus type I achalasia (Table 2; Figures 3(c) and 3(d)).

IL-13 has similar functions to IL-4. However, it also regulates type I collagen gene and thus participates in fibrosis. Type II achalasia patients showed an increase in IL-13-producing cells when compared with control tissue and types I and III achalasia patients (Table 2; Figures 3(e) and 3(f)).

### 3.6. Regulatory Cells

**Figure 4:**
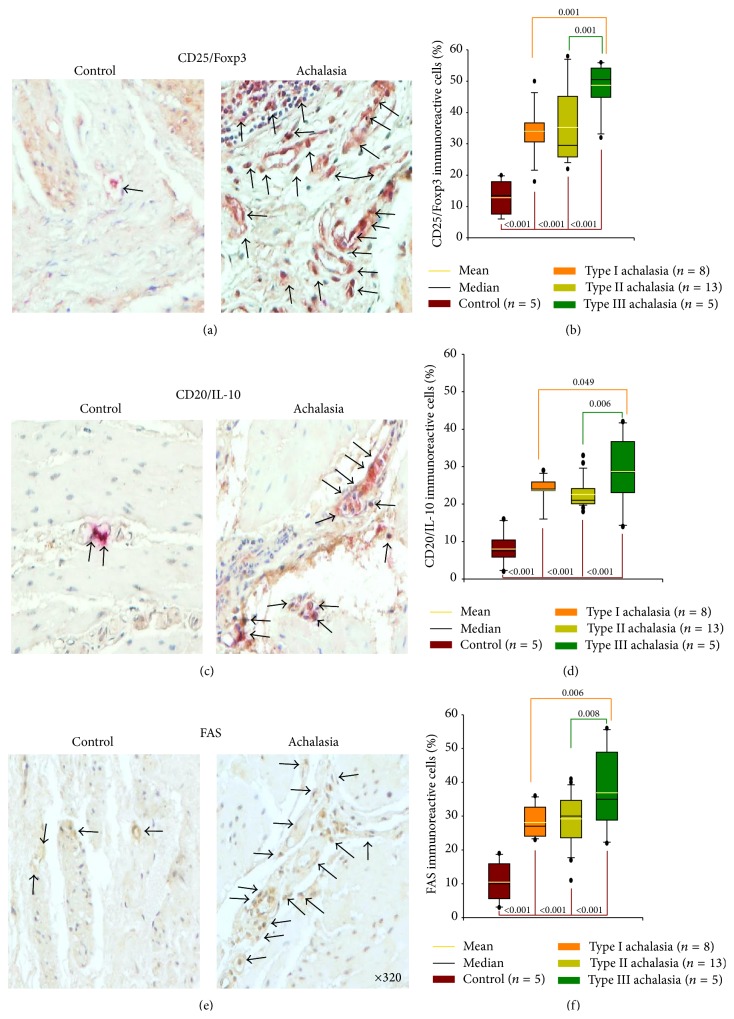
Regulatory cells and apoptosis in achalasia. Immunohistochemistry for (a) CD25/Foxp3 T cells, (c) CD20/IL-10 B cells, and (e) FAS cells in control and achalasia sections. Arrows depict immunoreactive cells. Original magnification was ×320. Results are expressed as mean (yellow line), median (black line), and 5th/95th percentiles of (b) CD25/Foxp3^+^ T cells, (d) CD20/IL-10^+^ B cells, and (f) FAS^+^ cells.

Regulatory T cells (Tregs) are a specialized subpopulation of T cells that suppress the activation of effector T cells. They maintain homeostasis and promote tolerance to self-antigens. Achalasia patients showed a higher Treg frequency in myenteric plexus of esophageal tissue compared with tissue controls. Nonetheless, the highest Treg cell percentage was found in type III achalasia versus types I and II (Table 2; Figures 4(a) and 4(b)).

Recently, the presence of a B cell subpopulation with regulatory capacity (Bregs) has been shown in humans. This suppressive function has been attributed to IL-10 production. Furthermore, Bregs promote T-cell differentiation to a regulatory phenotype and induce remission of autoimmune manifestations in murine models. The myenteric plexus of esophageal biopsies obtained from achalasia patients had a higher relative IL-10-producing B-cell percentage when compared with tissues from control group. It is noteworthy that type III achalasia patients showed a statistically significant increase levels versus types I and II achalasia (Table 2; Figures 4(c) and 4(d)).

### 3.7. Fas Expression

FAS receptor, also known as apoptosis antigen 1 (CD95), is a 36 KDa surface protein with a cytoplasmic domain of “cell death.” Its binding to FAS ligand induces complete apoptosis. The myenteric plexus of esophageal biopsies from achalasia patients had higher Fas-expressing cell frequency versus control tissue. Type III achalasia patients had highest Fas^+^ cell percentage when compared with types II and I achalasia (Table 2; Figures 4(e) and 4(f)).

### 3.8. Percentage of Circulating CD4^+^ T-Cell Subpopulations

**Figure 5:**
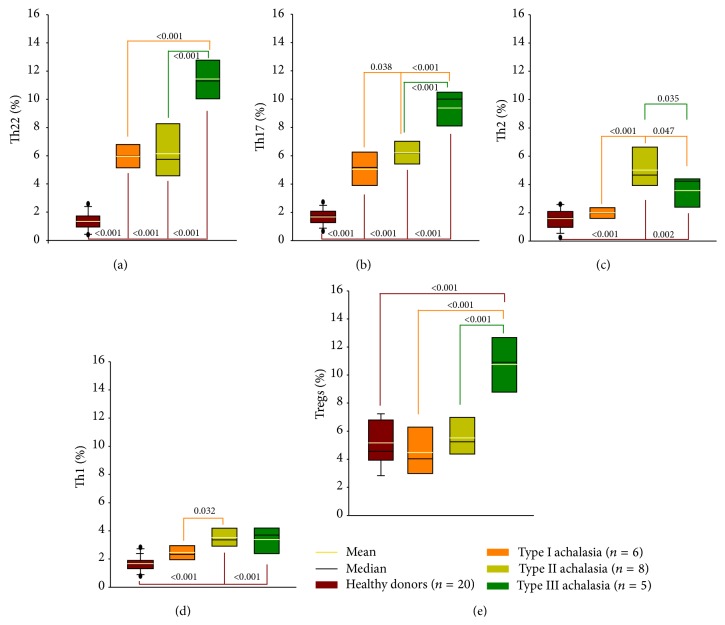
Circulating CD4^+^ T-cell subsets in achalasia. Relative percentage of circulating (a) Th22, (b) Th17, (c) Th2, (d) Th1, and (e) regulatory T cells. Results are expressed as mean (yellow line), median (black line), and 5th/95th percentiles of T cells.

To determine the different T-cell subpopulations, PBMCs were immunophenotyped and analyzed by flow cytometry. Relative percentage of circulating Th22 and Th17 cells from achalasia patients was higher when compared with healthy individuals. Type III achalasia patients showed an increment versus type I and II achalasia patients (Table 3; Figures 5(a) and 5(b)).

Regarding the Th2 cells, there was a significant increase in type III achalasia patients versus type I (Table 3; Figure 5(c)).

On the other hand, relative percentage of Th1 cells was higher in type II achalasia patients when compared with type I (Table 3; Figure 5(d)).

Finally, Tregs showed a statistically significant increase only in type III achalasia patients (Table 3; Figure 5(e)).

### 3.9. Antibodies against Myenteric Plexus

**Figure 6:**
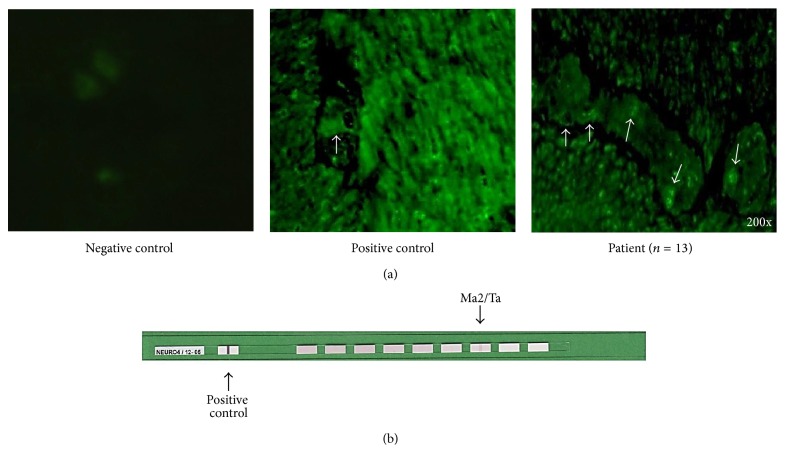
Anti-myenteric plexus antibodies and antigenicity. (a) Indirect immunofluorescence of monkey intestinal tissue containing Auerbach's plexus. Arrows show positive fluorescent nuclei and cytoplasm of cells that were recognized by circulating anti-myenteric plexus autoantibodies from achalasia patients (right panel). (b) Profile of neuronal antigens (antigens on membrane strips). Arrow in upper strip shows circulating autoantibodies against PNMA2 (Ma2/Ta) antigen. Arrow in lower strip shows circulating antibodies against recoverin antigen.

The prevalence of nuclear or cytoplasmic circulating autoantibodies against myenteric plexus in the sera from idiopathic achalasia patients was 100% (14/14) compared with healthy donors (0%) (Figure 6(a)). Most antibodies were positive against nuclei and nucleolus in myenteric plexus (Figure 6(a)).

### 3.10. Immunoblot Analysis

By immunoblot analysis, it was determined that 69% (9/13) of sera were positive to PNMA2 (Ma-2/Ta) antigen. Only one serum was labelled to recoverin (Figure 6(b)).

### 3.11. *In Situ* Viral Infection and Active Replication of HSV-1

**Figure 7:**
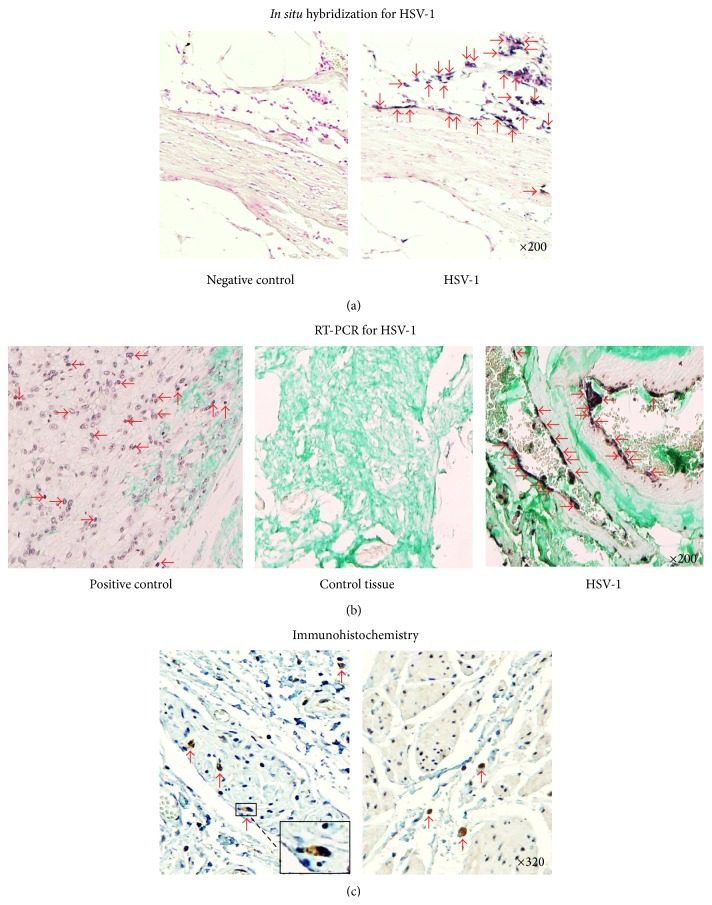
HSV-1 identification. (a)* In situ* hybridization. Arrows depict achalasia tissue with viral infection. (b)* In situ* RT-PCR for active replication of HSV-1. Arrows show antigen detection of HSV-1 infected cells in a skin lesion (positive control) and achalasia tissue. Original magnification was ×200. (c) Immunohistochemistry for antigen HSV-1 detection. Arrows depict immunoreactive cells. Original magnification was ×320.

HSV-1 DNA (viral infection), RNA (active replication), and virus were detected in all tissues (14/14) from achalasia patients by PCR (Figure 7(a)), RT-PCR (Figure 7(b)), and immunohistochemistry (Figure 7(c)), but not in control tissues. It is noteworthy that only one (1/14) and two (2/14) achalasia patients were cytomegalovirus- and Epstein-Barr virus-positive, respectively (*data not shown*).

## 4. Discussion

Idiopathic achalasia is an inflammatory disease caused by the loss of the inhibitory neurons of the esophageal myenteric plexus. Etiology of the myenteric plexus inflammation is still unknown [23]. Nevertheless, based on the evidence obtained in our research, it is possible to postulate an etio- and physiopathogenic mechanism for achalasia.

Initial event that triggers disease appears to be the result of a repetitive insult produced by neurotropic virus infection, likely HSV-1 [12, 13, 24–26], which induces a conspicuous and persistent inflammation at the perineural level, in Auerbach's plexus. Not all infected patients develop achalasia. Thus, it is not preposterous to suggest that only those individuals with genetic predisposition to develop a chronic autoinflammatory response will progress to the disease [13]. In fact, there are many studies that showed a positive association for the class II HLA antigen, including DQw1, DQA1^*∗*^0103, DQB1^*∗*^0601, DQB1^*∗*^0602, DQB1^*∗*^0603, DQB1^*∗*^0601, DQB1^*∗*^0502, and DQB1^*∗*^0503 alleles in Caucasians [4, 17, 27–29]. Furthermore, it has been demonstrated in 1,068 cases of achalasia from central Europe, Spain, and Italy that an eight-residue insertion at position 227–234 in the cytoplasmic tail of HLA-DQ*β*1 (encoded by HLA-DQB1^*∗*^05:03 and HLA-DQB1^*∗*^06:01) confers the strongest risk for achalasia. In addition, two amino acid substitutions in the extracellular domain of HLA-DQ*α*1 at position 41 (lysine encoded by HLA-DQA1^*∗*^01:03) and of HLA-DQ*β*1 at position 45 (glutamic acid encoded by HLA-DQB1^*∗*^03:01 and HLA-DQB1^*∗*^03:04) independently confer achalasia risk [29].

In addition, destruction of inhibitory ganglionic cells due to the abundant autoimmune inflammatory infiltrates, hypertrophy, and neuronal fibrosis during disease progression is found [30]. In most of the cases, pathology is accompanied by the presence of neuronal autoantibodies [18, 21] that contribute to the destruction of the myenteric plexus [31, 32] as previously has been demonstrated by Bruley des Varannes et al., where serum from achalasia patients but not from gastroesophageal reflux disease can induce phenotypic and functional changes in myenteric neurons which reproduce the characteristics of the disease and discard the fact that these autoantibodies could be an epiphenomenon [2].

In this study we determined* in situ* viral infection and active replication of HSV-1 but not CMV or EBV (*data not shown*) along myenteric plexus. HSV-1 preferential ports of entry are represented by the perioral and the esophageal mucosa. Furthermore, herpes viruses exhibit a strong tropism for nerve fibers and following the primary exposure, the viruses remain in a latent form in the nucleus of neurons [12]. Thus, one of the potential triggers for the development of immune-mediated diseases is an infection. In particular, in autoimmune diseases, characterized by immune-mediated inflammation to self-antigens, it has been established that viral or bacterial infections may trigger the cascade of events in genetic susceptible patients. Viral infection induce an increase in the amount of T-cell population infiltrates when compared with the controls and healthy donors, locally (biopsies) and systemically (circulating cells). To our knowledge, this is the first study that evaluates the presence of two CD4 T-cell subpopulations involved in autoimmune inflammation, Th22 and Th17 cells. Th17 cells synthesize IL-17A, IL-17F, IL-21, and so forth and have been demonstrated in autoimmune disease such as Sjögren's syndrome, multiple sclerosis, psoriasis, autoimmune uveitis, juvenile diabetes, rheumatoid arthritis, and Crohn's disease [15, 16, 20, 33, 34]. On the other hand, Th22 synthesizes primarily IL-22. It plays an important role in the pathogenesis of many autoimmune disorders, such as psoriasis, rheumatoid arthritis, Sjögren's syndrome, hepatitis, graft versus host disease, and allergic diseases [16, 35]. IL-17A-and IL-22-producing cells were the main cellular components of the inflammatory foci in achalasia tissue. In addition, an increase in relative percentage of circulating Th17 and Th22 cells was observed in achalasia patients when compared with healthy controls. These data strongly suggest that achalasia is also an autoimmune disease. Moreover, four of our achalasia patients had other autoimmune diseases such as Sjögren's syndrome (2), ankylosing spondylitis (1), and Guillain-Barre syndrome (1) suggesting that achalasia has an autoimmune component [4]. Finally, it has been reported that a severe recurrent achalasia cardia patient who responded to treatment of autoimmune acquired hemophilia with high-dose steroid therapy administered for 7 months without recurrence of the achalasia for more than 2 years. Thus, response of the achalasia to immunosuppressive therapy also suggests that achalasia is an autoimmune disorder [36].

Regarding types of achalasia, we hypothesized (a) that type III could be a more aggressive disease, (b) that type II achalasia is an earlier stage of a continuum natural history of the disease that evolves into type I, whereas type III could represent the earliest stage of the disease, or (c) that type III could be a disease with different physiopathology [5, 37]. In this context, we determine that achalasia type II has an increment of IL-22, IL-17A, Th1 cell percentage and IL-4- and IL-13-producing cells (potentially anti-inflammatory and profibrogenic cytokines, resp.) when compared with type I achalasia. This supports that type II achalasia is an active pathologic process in an earlier stage of the disease. Whereas* in situ* IL-17A-, CD25/Foxp3-, CD20/IL-10-, and FAS-producing cells and Th22, Th17, and Tregs peripheral cells are significantly higher in type III achalasia when compared with the other groups. Moreover, the concomitant presence of inflammatory (IL-17, IL-22) and regulatory cells (Tregs and IL-10-producing B cells) suggests that the proinflammatory and regulatory balance favors the former with the incapacity of the latter in trying to maintain homeostasis. Conversely the, as yet unknown, persistent inflammatory trigger surpasses the regulatory potential of Foxp3 T cells and CD20/IL-10 B cells, conducting to a more vigorous tissue damage characterized by an increase of MMP-9 and TIMP-1.

Finally, significant increase of autoantibodies against myenteric plexus in all the patients with achalasia also supports the hypothesis of an autoimmune mechanism. In order to characterize protein targets of circulating anti-myenteric autoantibodies, we used immunoblot analysis on primate cerebellum and myenteric plexus protein extract to establish whether achalasia sera with nuclear reactivity bind to proteins of a given molecular weight. A 69% of sera reacted with PNMA2/Ta (Ma2/Ta) and 8% reacted with recoverin antigen (Figure 6). To our knowledge, this is the first study that describes the specificity of anti-myenteric autoantibodies to Ma2/Ta protein, which are related to Sjögren's syndrome. However, we do not discard the possibility of the presence of other autoantibodies with different antigenic specificity, for example, GAD 65 [18].

There are potential limitations to the current study. First, studies of HLA association in Mexican Mestizo patients are needed for a better understanding of the disease. Second, this work was a cross-sectional study. Thus, longitudinal studies are required to provide the basis for the search of specific biomarkers that contribute to early diagnosis of the disease [38].

**Figure 8:**
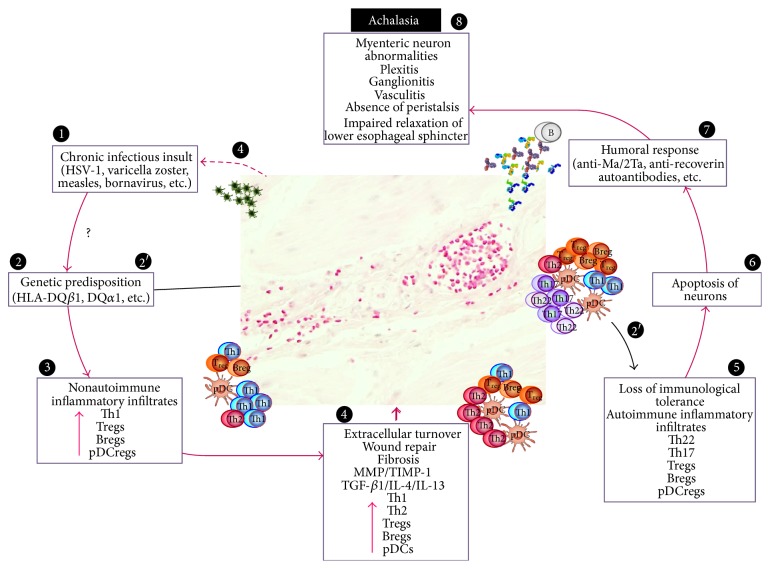
Proposed model of achalasia pathophysiology. (1) Active or latent infectious insult in achalasia is strongly suggested by several studies. Some neurotropic viruses such as the herpes family of viruses have predilection for squamous epithelium and may cause ganglion cell damage that is limited to the esophagus. (2) Some individuals with genetic predisposition will develop an aggressive inflammatory response. (3) At very early stage of the disease, it is possible that inflammatory infiltrates may be predominantly composed of Th1, Th2, and regulatory cell subsets (Tregs, Bregs, and plasmacytoid dendritic cells (pDCs)). (4) Repair of tissue after injury requires orchestrated coordination of several cell types and biosynthetic processes and is coordinated by an interacting group of pro- and anti-inflammatory cytokines, fibrous extracellular matrix (ECM) proteins to replace lost or damaged tissue, and products of metabolism such as oxygen radicals. The most prominent profibrogenic cytokines are TGF*β*, IL-4, and IL-13. ECM also mediates cellular crosstalk and does so in two ways. Newly deposited ECM is then rebuilding over time to emulate normal tissue. Matrix proteinases and their inhibitors (TIMPs) also are important, both during wound repair, tissue remodeling, and fibrosis. (2′, 5) If steps 1–4 happen repeatedly, chronic infection, only those individuals with genetic predisposition to develop a long-lasting autoinflammatory response will progress to develop the disease (loss of peripheral tolerance). Thus, autoinflammatory infiltrates are predominantly composed of Th22, Th17, and regulatory subpopulations. (6) Degeneration and significant loss of nerve fibers, associated with autoinflammatory infiltrates of the myenteric plexus, provide evidence of an immune mediated destruction of the inhibitory neurons, not only by necrosis but also by apoptosis (Fas/FasL overexpression). (7) Autoimmune etiology of achalasia is further supported by the presence of anti-myenteric autoantibodies in sera. (8) Pathophysiologically, achalasia is cause by autoinflammation, degeneration of nerves in the esophagus, plexitis, abnormalities in microvasculature, ganglionitis, and finally by the loss of inhibitory ganglion in the myenteric plexus.

Summing up, our results suggest that achalasia is a disease with an important local inflammatory and systemic autoimmune component [35]. This autoimmune response appears to be associated with the presence of HSV-1 and related to the high prevalence of specific autoantibodies against the myenteric plexus (Figure 8).

Our results shed further light into the preponderant autoimmune inflammation and the possible role of viral infection and certainly deserve to be studied in depth in order to evaluate the nature of genetic predisposition to the disease development. Our data may contribute to the identification of important disease cell and cytokine targets in achalasia, which finally may result in improved therapeutic management, for example, immunosuppressive therapy. Moreover, our findings may provide the basis for the search of specific biomarkers that contribute to early diagnosis of the disease.

## Significance of This Study

*What Is Already Known on This Subject?*

- Idiopathic achalasia is a rare esophageal motor disorder characterized by loss of myenteric plexus. Achalasia is postulated to be an autoimmune disease.
- The presence of circulating anti-myenteric autoantibodies has been reported.
- Some studies have implicated viral agents in the etiology and pathogenesis of the disease

*What Are the New Findings?*

- An increased percentage of two CD4^+^ T-cell subpopulations related to autoimmune diseases, Th17 and Th22, were identified in peripheral cells and their cytokines were detected in the inflammatory infiltrates of the lower esophageal sphincter biopsies from achalasia patients.
- Circulating anti-myenteric autoantibodies, predominantly directed against PNMA2 (Ma-2/Ta) antigen, were detected in sera from achalasia patients.
- HSV-1 DNA (viral infection), RNA (active replication), and virus were detected in the lower esophageal sphincter biopsies from achalasia patients.

*How Might It Impact on Clinical Practice in the Foreseeable Future?*

- Our data may contribute to the identification of important disease cell and cytokine targets in achalasia, which finally may result in improved therapeutic management, for example, immunosuppressive therapy.
- Our findings may provide the basis for the search of specific biomarkers that contribute to early diagnosis of the disease.

## Supplementary Material

Supplementary Figure 1. Flowchart of sample analysis.

## Figures and Tables

**Table 1:** Demographic, clinical, and laboratory variables.

	Blood healthy donors	Tissue control group	Type I achalasia	Type II achalasia	Type III achalasia
	(*n* = 20)	(*n* = 5)	(*n* = 8)	(*n* = 19)	(*n* = 5)
Age (years), mean ± SD	40.8 ± 10.1	72.2 ± 8.9^∗^	44.2 ± 14.2	45.6 ± 16.3	45.0 ± 17.4
Sex, female/male	12/8	4/1	4/4	12/7	4/1
Disease evolution (months), mean ± SD	ND	ND	43.7 ± 12.2	36.9 ± 7.9	69.0 ± 35.8
Dysphagia (%)	ND	ND	100	95	100
Regurgitation (%)	ND	ND	86	94	80
Weight loss (%)	ND	ND	71	78	100
Pyrosis (%)	ND	ND	57	42	40
Other autoimmune diseases (%)	ND	ND	0	11	40^∗^
ESR >20 mmHg (%)	ND	ND	17	25	20

^∗^ *P* < 0.05; ND: Not determined.

**Table 2:** Percentage of immunoreactive cells in achalasia patients.

	Control group	Type I achalasia	Type II achalasia	Type III achalasia
	(*n* = 5)	(*n* = 8)	(*n* = 13)	(*n* = 5)
*Extracellular matrix turnover proteins *				
MMP-9 (%)				
Mean ± SEM	13.6 ± 2.4	31.8 ± 2.2^∗∗∗^	38.5 ± 0.9^∗∗∗^	41.0 ± 1.5^∗∗∗^
Median	14.5	29.0	38.5	40.5
Range	2–25	23–45	29–47	35–48
TIMP-1 (%)				
Mean ± SEM	14.1 ± 1.5	25.9 ± 2.4^∗∗∗^	34.8 ± 1.6^∗∗∗^	35.1 ± 4.4^∗∗∗^
Median	13.0	24.0	35.5	40
Range	5–22	13–40	21–46	18–50

*Proinflammatory cytokines *				
IL-22 (%)				
Mean ± SEM	5.4 ± 1.1	27.0 ± 1.3^∗∗∗^	35.6 ± 1.6^∗∗∗^	39.7 ± 2.1^∗∗∗^
Median	5.0	28.5	38.0	40.5
Range	2–14	17–32	27–48	31–52
IL-17A (%)				
Mean ± SEM	4.7 ± 0.8	19.5 ± 1.5^∗∗∗^	22.8 ± 0.6^∗∗∗^	27.3 ± 1.3^∗∗∗^
Median	5.0	19.5	22.0	26.5
Range	1–8	11–29	19–31	22–35
CD4/IFN-*γ* (%)				
Mean ± SEM	3.8 ± 0.6	23.5 ± 1.1^∗∗∗^	28.0 ± 0.7^∗∗∗^	26.8 ± 0.7^∗∗∗^
Median	3.5	24.5	27.0	27.0
Range	2–8	16–29	22–36	23–30

*Profibrogenic cytokines *				
TGF-*β*1 (%)				
Mean ± SEM	7.0 ± 0.9	33.1 ± 1.4^∗∗∗^	27.9 ± 1.1^∗∗∗^	32.7 ± 1.5^∗∗∗^
Median	7.0	34.0	28.0	33.5
Range	3–11	21–40	16–38	25–40
IL-4 (%)				
Mean ± SEM	10.9 ± 1.6	21.2 ± 2.2^∗∗∗^	32.8 ± 1.0^∗∗∗^	31.4 ± 0.9^∗∗∗^
Median	11.0	18.0	33.0	32.0
Range	4–19	14–36	24–39	27–35
IL-13 (%)				
Mean ± SEM	6.4 ± 1.2	13.7 ± 0.5^∗∗∗^	20.0 ± 1.1^∗∗∗^	15.9 ± 0.6^∗∗∗^
Median	6.5	13.5	21.0	15.5
Range	1–11	11–16	9–27	13–19

*Regulatory cells *				
CD4/Foxp3 (%)				
Mean ± SEM	12.8 ± 1.6	34.0 ± 2.1^∗∗∗^	35.2 ± 2.6^∗∗∗^	48.7 ± 2.3^∗∗∗^
Median	13.5	34.0	29.5	50.5
Range	6–20	18–50	22–58	32–56
CD20/IL-10 (%)				
Mean ± SEM	8.0 ± 1.2	23.7 ± 1.1^∗∗∗^	22.6 ± 0.8^∗∗∗^	28.7 ± 2.9^∗∗∗^
Median	7.5	24.0	21.0	28.5
Range	2–16	16–29	18–33	14–42

*Apoptosis *				
FAS (%)				
Mean ± SEM	10.5 ± 1.7	28.1 ± 1.3^∗∗∗^	29.2 ± 1.5^∗∗∗^	38.3 ± 3.7^∗∗∗^
Median	10	27.0	30.0	35.0
Range	3–19	23–36	11–41	22–56

^∗^ *P* < 0.05 control versus achalasia patients.

^∗∗^ *P* < 0.01 control versus achalasia patients.

^∗∗∗^ *P* < 0.001 control versus achalasia patients.

**Table 3:** Percentage of Th22-, Th17-, Th2-, Th1-, and Tregs-circulating cells in achalasia patients.

	Healthy donors	Type I achalasia	Type II achalasia	Type III achalasia
	(*n* = 20)	(*n* = 6)	(*n* = 8)	(*n* = 5)
*IL-22-expressing T cells* *(Th22 %) *				
CD3^+^/CD4^+^/CD161^−^/IL-22^+^				
Mean ± SEM	1.3 ± 0.1	6.4 ± 0.7^∗∗∗^	5.9 ± 0.9^∗∗∗^	11.4 ± 0.7^∗∗∗^
Median	1.4	6.1	5.7	11.3
Range	0.4–2.7	5.1–8.9	3.3–9.4	9.5–13.8

*IL-17A-expressing T cells* *(Th17 %) *				
CD3^+^/CD4^+^/CD161^+^/IL-17A^+^				
Mean ± SEM	1.6 ± 0.1	4.2 ± 0.1^∗∗∗^	6.2 ± 0.6^∗∗∗^	9.4 ± 0.6^∗∗∗^
Median	1.7	4.1	6.4	10.0
Range	0.6–2.7	3.8–6.7	4.0–8.2	7.2–10.6

*IL-4-expressing T cells* *(Th2 %) *				
CD3^+^/CD4^+^/CD14^−^/IL-4^+^				
Mean ± SEM	1.6 ± 0.2	2.3 ± 1.1	5.0 ± 0.5^∗∗∗^	3.6 ± 0.5^∗∗∗^
Median	1.6	2.5	4.7	4.2
Range	0.2–2.6	1.5–7.83	3.8–7.2	2.0–4.6

*IFN-γ-expressing T cells* *(Th1 %) *				
CD3^+^/CD4^+^/CD14^−^/IFN-*γ* ^+^				
Mean ± SEM	1.7 ± 0.1	2.8 ± 0.5	3.4 ± 0.3^∗∗∗^	3.4 ± 0.5^∗∗∗^
Median	1.7	2.6	3.2	3.7
Range	0.7–3.0	1.8–4.2	2.7–4.6	2.1–4.7

*Foxp3-expressing T cells* *(Tregs %) *				
CD3^+^/CD4^+^/CD25^hi^/Foxp3^+^				
Mean ± SEM	5.2 ± 0.4	4.3 ± 0.6	5.9 ± 0.6	10.7 ± 0.9^∗∗∗^
Median	4.6	4.2	6.1	10.8
Range	2.3–7.3	2.5–6.2	3.7–7.2	7.7–12.8

^∗^ *P* < 0.05 control versus achalasia patients.

^∗∗^ *P* < 0.01 control versus achalasia patients.

^∗∗∗^ *P* < 0.001 control versus achalasia patients.

## Abbreviations

AP: Alkaline phosphatase
APC: Allophycocyanin
Bregs: Regulatory B cells
CD: Cluster differentiation
cDNA: Complementary DNA
DAB: Diaminobenzidine
dNTPs: Deoxyribonucleotide triphosphate
DTT: Dithiothreitol
dUTP: Deoxyuridine triphosphate
ESR: Erythrocyte sedimentation rate
FITC: Fluorescein isothiocyanate
FMO: Fluorescence minus one
Foxp3: Forkhead box P3
HCV: Hepatitis C virus
HD: Healthy donors
HIV: Human immunodeficiency virus
HRM: High-resolution manometry
HRP: Horseradish peroxidase
HSV-1: Herpes simplex virus-1
IFN-*γ*: Interferon-gamma
IL: Interleukin
LES: Lower esophageal sphincter
LINK: Dextran polymer coupled with secondary antibodies against mouse and rabbit immunoglobulins
MgCl_2_: Magnesium chloride
M-MLV: Moloney murine Leukemia virus enzyme
MMP-9: Matrix metalloproteinase-9
PBMCs: Peripheral blood mononuclear cells
PCR: Polymerase chain reaction
PE: Phycoerythrin
PeCy5: Phycoerythrin cychrome 5
RT: Reverse transcription
SD: Standard deviation
SEM: Standard error of the mean
TGF-*β*1: Transforming growth factor-beta
Th: T helper cells
TIMP-1: Tissue inhibitor of metalloproteinase-1
Tregs: Regulatory T cells.
