# Supplementary material for: Achalasia—An Autoimmune Inflammatory Disease: A Cross-Sectional Study
Source: J Immunol Res. 2015 May 20;2015:729217. doi: 10.1155/2015/729217 (PMC4452860; doi:10.1155/2015/729217)
Supplement: Supplementary file 1 — Supplementary Figure 1. Flowchart of sample analysis. [file 729217.f1.pdf]

Supplementary Materials

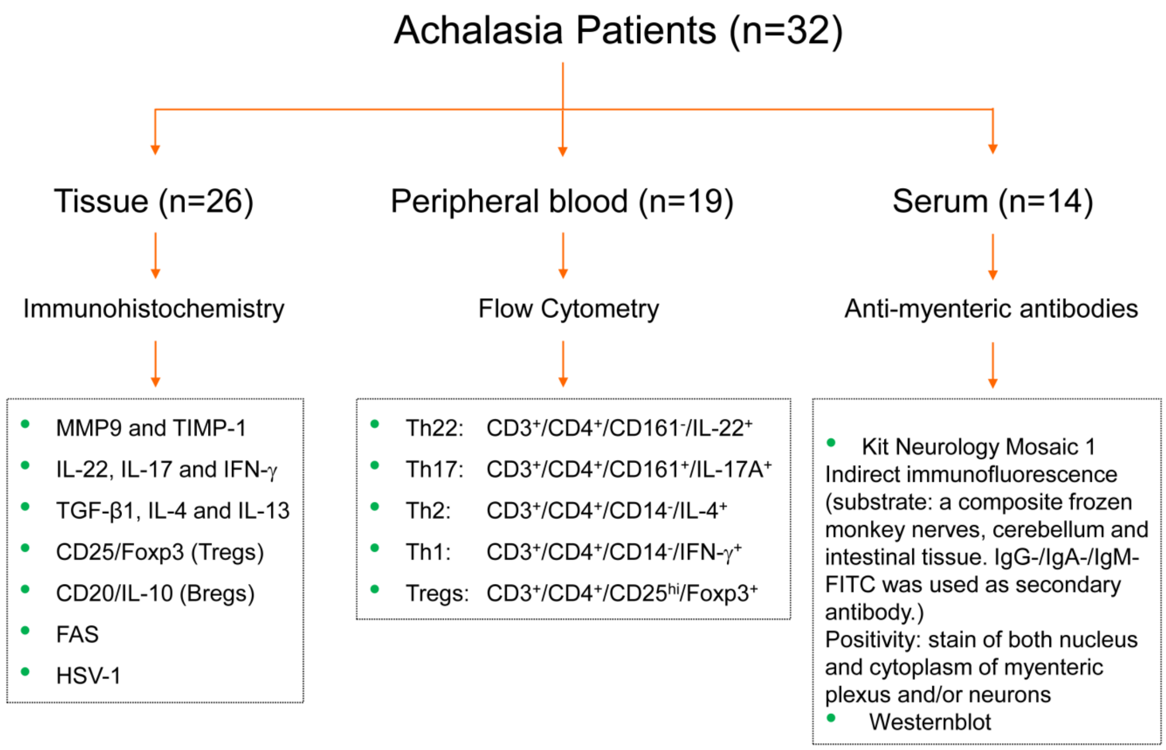

Statistical Analysis: One Way Analysis of Variance on Ranks. Holm-Sidak Method

Figure S1
